# Analysis of factors related to recurrence of paediatric hepatoblastoma - a single Centre retrospective study

**DOI:** 10.1186/s12887-019-1870-3

**Published:** 2019-12-10

**Authors:** Wei Yang, Yiwei Chen, Yijin Huang, Huanmin Wang

**Affiliations:** Department of Surgical Oncology, Beijing Children’s Hospital, Capital Medical University, National Center for Children’s Health, 100045, No.56 Nanlishi road, Beijing, China

**Keywords:** Hepatoblastoma, Alpha fetoprotein, Risk factors

## Abstract

**Background:**

This study was performed to identify risk factors associated with recurrence of hepatoblastoma.

**Methods:**

A retrospective study was conducted on 56 patients with hepatoblastoma from 2012 to 2015 in Beijing Children’s Hospital. Pretreatment extension stage (PRETEXT), serum alpha fetoprotein (AFP) value, change trend of tumors after treatment and some other clinical characteristics were collected and analyzed. The comparison of independent variables that were not distributed normally was performed with the log-rank test.

**Results:**

Twenty-eight patients with tumour recurrence and 28 patients without recurrence were included in this study, and the median age at presentation was 46.5 (26, 71.5) months. There was a significant difference in the 3-year recurrence-free survival (RFS) probability between patients aged over 54 months and those younger than 54 months (*p* = 0.007). After neoadjuvant chemotherapy, the chance of recurrence in partial response (PR) patients was significantly lower than that in stable disease (SD) patients (*p* = 0.004). The 3-year RFS rate of patients with a reduction in AFP of more than 60% after neoadjuvant chemotherapy was significantly higher than that of patients with a reduction of less than 60% (*p* = 0.005). The postoperative follow-up revealed that patients whose postoperative AFP fell to normal levels within 6 months of the start of treatment had a 3-year RFS rate of 68.6%, which is higher than that of patients whose AFP fell below the normal range after 6 months (*p* = 0.0005). Finally, the multivariate analysis by Cox regression showed that AFP decreased by less than 60% and tumour size decreased by less than 50% after neoadjuvant chemotherapy were significant independent prognostic risk factors for the 3-year RFS rate. The other clinical features were not significantly associated with tumour recurrence in this study.

**Conclusions:**

Through this study, we concluded that the prognosis of childhood HB is related to the age at presentation and the response of chemotherapy. The results of the multivariate analysis showed that AFP decreased by less than 60% and tumour size decreased by less than 50% after neoadjuvant chemotherapy were significant independent prognostic risk factors. These findings can be helpful to evaluate therapeutic effects and predict prognosis.

## Background

Hepatoblastoma (HB) is a rare type of primary malignant liver tumour that mostly occurs in infants and children under 3 years of age [1]. The pathological features of HB consist of tissues resembling foetal liver cells, mature hepatocytes or biliary cells [2]. The most common symptom is abdominal mass [3]. An important method of examination for HB is measuring the serum alpha-fetoprotein (AFP) value. The patient will be suspected of having HB if he or she has an obviously elevated AFP value. However, the prognosis of patients with HB is poor if AFP is not elevated at diagnosis [4, 5]. AFP is also used as a sensitive marker of successful treatment. If the tumour is completely removed, the AFP value will gradually drop to normal [6].

Currently, surgical tumour resection, chemotherapy and orthotopic liver transplantation are used for the treatment of HB [7, 8]. The survival of patients with HB relies largely on surgical resection [9, 10]. Experience has demonstrated that HBs respond well to chemotherapy and that cisplatin-based regimens result in good outcomes for these tumours. Some clinical research demonstrates that doxorubicin (if not given during initial treatment) and irinotecan are effective for relapsed and refractory tumours [11]. However, the long-term survival of patients with recurrence of refractory HB is relatively low, especially in patients with distant metastases [12, 13]. The treatment of relapsed patients still faces some challenge. Thus, it is important to identify relevant factors to predict the possibility and reduce the risk of recurrence.

## Methods

### Study subjects

A single-centre retrospective analysis of 56 patients with HB diagnosed in Beijing Children’s Hospital between June 2012 and September 2015 was performed to identify risk factors associated with prognosis. This study included 28 patients with relapsed HB (21 with local recurrence and 7 with pulmonary metastases) and 28 patients without recurrence who were selected by a random numbers table method after excluding some patients with missing data in the same period. The patients were diagnosed based on pathological findings and were assessed by serum AFP level and abdominal computed tomography (CT) or magnetic resonance imaging (MRI) with contrast enhancement. Distant metastases, such as lung metastases, were identified by positron emission tomography (PET)/ CT or CT scan. The patients were categorized into four stages based on the PRETEXT staging system adopted by the International Childhood Liver Tumours Strategy Group (SIOPEL) [14]. Furthermore, these cases were stratified into standard-risk and high-risk groups according to additional criteria.

### Treatment

For PRETEXT II, III and IV cases, 4 cycles of neoadjuvant chemotherapy were adopted after pathological confirmation via core needle biopsy. Neoadjuvant chemotherapy consisted of C5V (20 mg/m^2^ cisplatin on days 1–5, 300 mg/m^2^ fluorouracil on days 1–2, 1.5 mg/m^2^ vincristine on day 2) for the standard-risk group and C5VD (20 mg/m^2^ cisplatin on days 1–5, 300 mg/m^2^ fluorouracil on days 1–2, 1.5 mg/m^2^ vincristine on day 2, 25 mg/m^2^ pirarubicin on days 1–3) or PLADO (20 mg/m^2^ cisplatin on days 1–5, 25 mg/m^2^ pirarubicin on days 1–3) for the high-risk group. Then, the patients underwent tumourectomy. After surgery, patients underwent 4–8 cycles of chemotherapy. Adjuvant chemotherapy regimens consisted of C5V (20 mg/m^2^ cisplatin on days 1–5, 300 mg/m^2^ fluorouracil on days 1–2, 1.5 mg/m^2^ vincristine on day 2) for the standard-risk group and C5VD (cisplatin on days 1–5, 300 mg/m^2^, fluorouracil on days 1–2, 1.5 mg/m^2^ vincristine on day 2, 25 mg/m^2^ pirarubicin on days 1–3) or cumulative cisplatin dose (CCD) (80 mg/m^2^ cisplatin on day 1, alternating every 2 weeks with 500 mg/m^2^ carboplatin on days 1–2 plus 30 mg/m^2^ doxorubicin on days 1–2).

### Clinical evaluation

We evaluated each patient using a post-treatment extension (POSTTEXT) system and analysed the serum AFP value using laboratory tests through the laboratory of Beijing Children’s Hospital after neoadjuvant chemotherapy and after surgery. It was recorded as ng/ml (normal range, 0–8 ng/ml). Moreover, the time when the AFP value dropped to normal was recorded. Furthermore, according to the evaluation criteria of the World Health Organization (WHO) on solid tumour treatment effect, we measured two major vertical diameters and calculated their product before and after neoadjuvant chemotherapy to evaluate the change trend of tumour size. The evaluation of the response of objective lesions was performed as follows: complete response (CR), disappearance of all target lesions; partial response (PR), at least a 50% decrease in the product of the major vertical diameters of target lesions; stable disease (SD), decrease in the product of major vertical diameters of the target lesions less than 50% or an increase of more than 25%; and progressive disease (PD), a 25% increase in the product of the major vertical diameters of the target lesions or the appearance of new lesions [15]. Other clinical characteristics were collected and analysed retrospectively, including age at presentation, sex, pathological subtype, initial AFP values, surgical strategy, and residual tumour. The purpose was to identify confounding factors.

### Follow-up

Patients were followed up every three months until August 2018 by email and telephone.

We investigated the patient survival, recurrence and progression and the value of serum AFP.

We defined recurrence as both local recurrence and distant metastasis, which were annotated separately in Table 1.

**Table 1:** clinical characteristics of HBs associated to prognoses

Variables		Results	3-year RFS Rate	*P*
Age (median, months)		46.5 (26,71.5)		
≥54 months (n, %)	YES	37(66)	63.9%	0.007
	No	19(34)	25%	
PRETEXT (n, %)				0.089
	I	0	0	
	II	26(46)	65.4%	
	III	26(46)	38.5%	
	IV	4(8)	25%	
SIOPEL risk stratification (n, %)				0.239
	SR	27(48)	59.9%	
	HR	29(52)	40.1%	
Tumor size (median, cm^2^)		100.84 (69.76,142.31)		
Initial serum AFP (median, ng/ml)		31,125 (5434,74,250)		
Non-recurrent (n)		28		
Recurrent (n)		28		
Site of tumour recurrence (n, %)	Local	21(75)		
	Lung metastasis	7(25)		
Prognoses (n, %)	Survive	46(82)		
	Death	7(13)		
	Lost to follow up	3(5)		
follow-up time (median, months)		44		

“Tumor size” -two major vertical diameters product; “*PRETEXT*”- Pretreatment extension stage; “*AFP*”- alpha-fetoprotein; “*SR*”- standard risk; “*HR*”- high risk; “*RFS*”- recurrence free survival

### Statistical analysis

Survival was calculated by using the Kaplan-Meier method with a confidence interval (CI) of 95%. Receiver operating characteristic (ROC) curve analysis was performed to determine the cut-off values and to analyse factors relevant to tumour recurrence. The comparison of independent variables that not distributed normally was performed with the log-rank test. SPSS software version 20.0 was used for statistical analysis. *P* < 0.05 was considered to represent a significant difference.

The current study was approved by the Committee on Human Study of the Beijing Children’s Hospital and the Ethics Review Committee of Beijing Children’s Hospital (No. 2019-k-11).

## Results

### Treatment and response

**Fig. 1:**
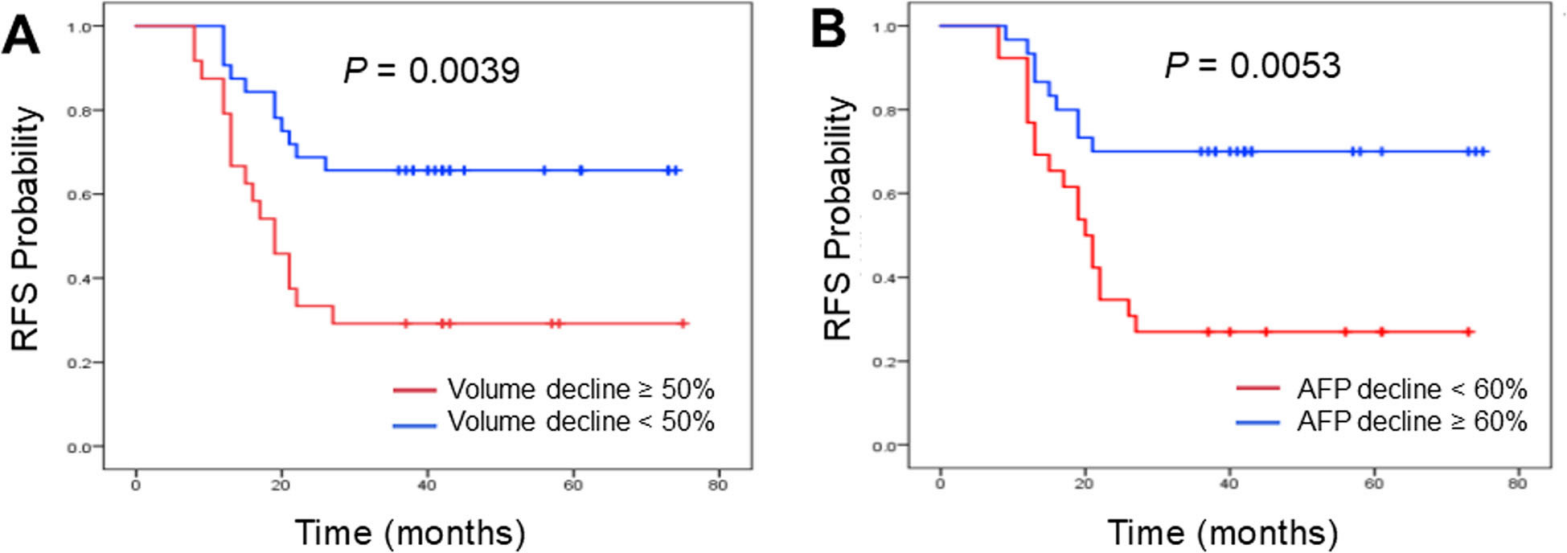
**a**: Relationship between the decline of tumor volume and 3 year RFS in this study. **b**: Relationship between the decline of serum AFP and 3 year RFS in this study

A total of 56 patients (32 male and 24 female) were included in this study. Twenty-eight patients experienced tumour recurrence after surgery. Among them, 21 (75%) patients had local recurrence, and 7 (25%) had lung metastases. Follow up was performed until August 2018, with a median follow-up time of 44 months. Seven of the patients died due to tumour recurrence or progression, 46 patients survived and 3 were lost to follow-up. The 3-year overall survival (OS) rate was 72% for the recurrence patients and 100% for the non-recurrence patients. Thirty-five patients with standard-risk HB received neoadjuvant chemotherapy of C5V, 17 with high-risk HB received C5VD, and 4 with high-risk HB received PLADO for 4 cycles before surgical tumour resection was performed. After induction chemotherapy, the product of the median of the major vertical diameters decline proportion was 52.5 (40.5, 60.75) %. According to the evaluation criteria of the WHO on solid tumour treatment effects, 32 patients were defined as achieving a PR, and 24 patients were defined as having SD; no patients had a CR or PD. The 3-year RFS rates of the PR and SD patients were 65.6 and 29.2%, respectively (Fig. 1). Based on the statistical analysis, the chance of recurrence after chemotherapy in PR patients was significantly lower than that in SD patients (*p* = 0.004) (Table 2).

**Table 2:** treatment and response of HBs associated to prognoses

Variables		Results	3-year RFS rate	*P*
AFP decreased after neoadjuvant chemotherapy (n, %)				0.005
	< 60%	26 (46)	28%	
	≥ 60%	30 (54)	68%	
Evaluation criteria of WHO (n, %)				0.004
	CR	0		
	PR	32 (57)	65.6%	
	SD	24 (43)	29.2%	
	PD	0		
POSTTEXT (n, %)				0.476
	I	9 (16)	66.7%	
	II	30 (54)	50.0%	
	III	17 (30)	41.1%	
	IV	0		
Surgical strategy (n, %)				0.747
	Left lobectomy	14 (25)	42.9%	
	Right lobectomy	33 (59)	54.6%	
	Extended hepatectomy	9 (16)	44.4%	
AFP fell to normal (n, %)				0.0005
	< 6 months	27 (48)	68.6%	
	≥ 6 months	29 (52)	50.0%	

“*POSTTEXT*”- Posttreatment extension stage; “*CR*”- complete response; “*PR*”- partial response; “*SD*”- stable disease; “*PD*”- progressive disease

After receiving 4 cycles of neoadjuvant chemotherapy, serum AFP decreased significantly in most patients before surgery. The AFP values of 10 of the patients fell back into the normal range. The 3-year RFS of patients with a decrease in AFP of more than 60% (68%) after neoadjuvant chemotherapy was significantly higher than that of patients with a decrease rate of less than 60% (28%) (*p* = 0.005) (Table 2) (Fig. 1). All patients were reassessed according to the posttreatment (POSTTEXT) staging system. There were 9 patients with POSTTEXT I, 30 with POSTTEXT II, and 17 with POSTTEXT III. There were no significant differences in 3-year RFS rate in terms of different POSTTEXT stages (*p* = 0.476). All patients underwent liver tumour resection, 14 underwent left lobectomy, 33 underwent right lobectomy and 9 underwent extended hepatectomy. The number of relapsed patients were 8/28, 15/28, and 5/28, respectively. There were no significant differences in the 3-year RFS rates among patients who underwent liver tumour resection with different surgical strategies (Table 2). The pathological reports showed that 45 (80%) patients had negative microscopic margins and 7 (13%) patients had positive microscopic margins, with 3-year RFS rates of 51.1 and 42.9%, respectively (*p* = 0.718). In the seven patients with positive margins, 4 had local tumour recurrence, and 3 had tumour-recurrence free.

Twenty-nine patients received the C5V chemotherapy regimen, 19 received C5VD and 8 received CCD for 6–8 cycles after operation. Postoperative follow-up revealed that 27(48%) patients whose postoperative AFP fell to normal levels within 6 months of the start of treatment had a three-year RFS of 68.6%, which is higher than that of patients whose AFP fell below the normal range after 6 months (50%) (*p* = 0.0005) (Table 2).

### Clinical characteristics

In this study, the median age at presentation was 46.5 (26, 71.5) months (range, 7 months to 141 months). There was a significant difference in the 3-year RFS probability between the patients over 54 months (25%) and those younger than 54 months (63.9%) (*p* = 0.007) (Table 1).

The patients were categorized based on 5 pathological subtypes: foetal, embryonal, foetal-embryonal mixed, macro-trabecular and small cell undifferentiated (SCU) based on the international paediatric liver tumour consensus classification of the Children’s Oncology Group (COG) [16]. The numbers of patients with each subtype were 2, 9, 35, 3, and 1, respectively. There were 6 patients for whom the pathological subtype was unable to be defined. Because the number of patients with SCU was small, it was not possible to conduct a survival analysis for each pathological subtype between children with recurrence and non-recurrence.

In this study, 26 (46%) patients were in PRETEXT II, 26 (46%) were in PRETEXT III and 4 (8%) were in PRETEXT IV based on the imaging assessment. The 3-year RFS rates differed among different PRETEXT stages, but the differences were not statistically significant (*p* = 0.089). Moreover, the PRETEXT annotation factors were documented. Among these patients, 26 (46%) had PRETEXT annotation factors (5 patients with “P1–3”; 8 with “V1–3”; 2 with “C”; 3 with “R” and 8 with “M”, no “E, F, N and H” criteria) *. The patients were classified into a standard-risk group (27/56, 48%), defined as PRETEXT I, II, or III, and a high-risk group (29/56, 52%), defined as PRETEXT IV or the presence of annotation factors based on the risk stratification recommend by SIOPEL (Table 1) [17]. The 3-year RFS rates differed between different risk groups, but the difference was not statistically significant (*p* = 0.239). After receiving neoadjuvant chemotherapy, the metastatic lesions of 6 patients with lung metastases at presentation disappeared before liver surgery. Among them, one patient developed pulmonary metastases again a few months after the end of the entire treatment. Two patients with lung metastases at presentation had isolated lung lesions less than 5 mm in diameter before hepatectomy, which disappeared after two cycles of postoperative chemotherapy and did not recur.

Among the 56 patients, the median initial serum AFP value was 31,125 (5434, 74,250) ng/ml (range, 1,330,000 ng/ml to 1778 ng/ml). The product of the two major vertical diameters of tumour lesions was calculated to estimate tumour size at the beginning of treatment, with a median of 100.84 (69.76,142.31) cm^2^ (range, 227.52 cm^2^ to 36 cm^2^). In the assessment of 3-year RFS, these two characteristics had no statistically significant difference in terms of tumour recurrence.

### Risk factors

Finally, multivariate analysis by Cox regression was performed. The results showed that AFP that decreased by less than 60% and tumour size that decreased by less than 50% after neoadjuvant chemotherapy were significant independent prognostic risk factors for 3-year RFS.

## Discussion

HB is the most common primary hepatic malignant tumour in children, with an annual incidence of 1.2–1.5 cases per million people [18]. The comprehensive treatment strategy of surgery combined with chemotherapy has greatly improved the overall prognosis of HB, but the disease is still life-threatening for some patients due to the recurrence or progression of the disease [19, 20]. This study focused on the factors related to HB recurrence and found the following risk factors in this study: patient age older than 54 months, AFP that decreased by less than 60% after the patient received neoadjuvant chemotherapy, tumour size that failed to achieve partial remission after neoadjuvant chemotherapy, and AFP that failed to return to a normal range within 6 months after receiving treatment.

HBs occur mostly in young children, especially in patients under three years of age. Age was not considered a separate risk factor in previous risk classifications recommended by the COG and SIOP. In this study, the probability of recurrence in children with an onset age older than 54 months was significantly higher. This finding suggested that older children with HB have a probability of poor prognosis. As a serum marker of HB, AFP is a sensitive indicator for determining a good response to chemotherapy. After chemotherapy, most patients will have a significant decline in AFP levels. This phenomenon has been studied and reported by many experts [21]. The purpose of this study was to find a specific point value to assess the relationship between prognosis and the trend of AFP decline after neoadjuvant chemotherapy. After analysis, it was shown that if the patient’s AFP value dropped by more than 60% after preoperative chemotherapy, they would have a reduced risk of tumour recurrence. A shrinking tumour size after chemotherapy is a characteristic manifestation in most malignant tumours. The reason for the shrinking tumour size is generally thought to be that chemotherapy drugs kill tumour cells, result in massive tumour tissue necrosis, and lead to tumour reduction. In this study, we used the two largest vertical diameters’ product to represent the tumour volume and compare the initial tumour size with that after chemotherapy. The results showed that after neoadjuvant chemotherapy, the two largest vertical diameter products of the tumour decreased by more than 50%; that is, patients who achieved PR according to the WHO evaluation criteria had a reduced risk of tumour recurrence. This reduction may be attributed to the shrinkage of tumours, resulting in better resectability and relieving other annotation risk factors, such as major vessel invasion. Monitoring the decrease in AFP after treatment indicated that an AFP value that failed to fall into a normal range within six months was a risk factor associated with recurrence. We hypothesized that tumours’ sensitivity to chemotherapy results in a significant decrease in serum AFP before surgery. This decrease results in a reduction in preoperative AFP load, a more effective clearance of tumour cells from the blood and the prevention of tumour cell proliferation, which lead serum AFP levels to return to normal more quickly after surgery. Compared to the patients who had a good respond to chemotherapy, the decrease in serum AFP was slower in patients with a worse response, confirming that active tumour cells remained, which may lead to recurrence after chemotherapy.

It has been widely accepted that the SCU histology is a risk factor for HBs [22]. Due to the low number of SCU patients in this group, a statistical analysis was not possible. In this study, there was no significant difference in the 3-year RFS rates between patients with microscopically negative margins and those with positive margins. There was no evidence of a definite relationship between microscopically positive margins and local recurrence in this study. In recent years, some experts have reported that a positive margin during surgery will not worsen the prognosis of hepatoblastoma [23]. We do not consider such patients to have a higher risk of recurrence if they are treated with a sufficient intensity and course of chemotherapy. According to the SIOPEL study, approximately 50% of lung metastases can achieve complete remission with preoperative chemotherapy [13]. This proportion was higher in this study. There was no correlation between postoperative pulmonary metastatic recurrence and lung metastases at presentation.

Other clinical factors were analysed retrospectively, including the surgical strategy, initial serum AFP value and tumour size at the beginning of therapy. However, no correlation was found with tumour recurrence. The results of a comparative study using PRETEXT or POSTTEXT stage, annotation factors, and SIOPEL risk stratification showed that there was a difference in 3-year RFS, but the difference was not statistically significant.

The limitation of this study is that it is a retrospective study with a small sample size. We plan to conduct a prospective evaluation and external verification to confirm our findings and supply the research content.

## Conclusions

Through this study, we concluded that the prognosis of childhood HB is related to the age at presentation and the response of chemotherapy. The results of the multivariate analysis showed that AFP that decreased by less than 60% and tumour size that decreased by less than 50% after neoadjuvant chemotherapy were significant independent prognostic risk factors. We should perform postoperative intensive chemotherapy for such patients and expand the sample for further validation. These findings can be helpful to evaluate therapeutic effects and predict prognosis.

## Data Availability

All data generated or analyzed during this study are included in this published article and its supplementary information files.

## Abbreviations

AFP: Serum Alpha Fetoprotein
C: Involvement of the caudate lobe
C5V: Cisplatin / 5-fluorouracil / Vincristine
C5VD: Cisplatin /5- fluorouracil / Vincristine
CCD: Cisplatin / Carboplatin / Doxorubicin
COG: Children’s Oncology Group
CR: Complete Response
CT: Computed Tomography
E: Extrahepatic tumour
F: Multifocal tumour
H: Tumour haemorrhage
HB: Hepatoblastoma
M: Distant metastasis
MRI: Magnetic Resonance Imaging
N: Positive lymph nodes
P: Macrovascular involvement of portal vein
PD: Progressive Disease
PET: Positron Emission Tomography
PLADO: Cisplatin / Pirarubicin
POSTTEXT: Post-Treatment Extension
PR: Partial Response
PRETEXT: PRE-treatment Extension
R: Tumour rupture
RFS: Recurrence-Free Survival
ROC: Receiver operating characteristic
SCU: Small Cell Undifferentiated
SD: Stable Disease
SIOPEL: Childhood Liver Tumours Strategy Group
V: Involvement of hepatic veins and vena cava
WHO: World Health Organization
